# The World Health Organization Coalition of Interested Parties Network: a unified and coordinated approach to regulatory system strengthening

**DOI:** 10.3389/fmed.2026.1725720

**Published:** 2026-02-24

**Authors:** Andrea Keyter, Tariro Sithole, Alireza Khadem Broojerdi, Jude Nwokike, Martin Harvey-Allchurch, Hiiti Sillo

**Affiliations:** 1 World Health Organization, Geneva, Switzerland; 2 World Health Organization Liaison Office to the African Union & UNECA, Addis Ababa, Ethiopia; 3 United States Pharmacopeial Convention, Maryland, MD, United States; 4 European Medicines Agency, Amsterdam, Netherlands

**Keywords:** coalition of interested parties (CIP) Network, global benchmarking tool (GBT), national regulatory authority (NRA), regulation of medical products, regulatory systems strengthening (RSS)

## Abstract

Strong and effective regulatory systems are essential for achieving universal health coverage and ensuring access to safe, effective and quality-assured medical products. Yet, many countries have not yet achieved a well-functioning, stable and integrated regulatory system, with capacity gaps continuing to hinder the performance of national regulatory authorities. In response, the World Health Organization developed the Five-Step Capacity Building Model to guide and strengthen regulatory systems and the Global Benchmarking Tool, and further established the Coalition of Interested Parties (CIP) Network as a global coordination mechanism to unify and optimize regulatory system strengthening support. This manuscript describes the development, operationalization and measurable impact of the CIP Network, which has grown to include 33 active members, including leading global donors, development agencies and technical partners. Between 2023 and 2025, the CIP Network facilitated over USD 235 million in targeted investment and coordinated nearly 600 technical activities across more than 50 countries. Anchored by WHO’s Global Benchmarking Tool and the implementation of Institutional Development Plans (IDPs), the Network operationalizes support through country-led coordination, shared visibility of priorities, continuous communication and accountability tracking. Member contributions span all regulatory functions, enabling rapid deployment of context-specific technical expertise. Regional and country-level case studies illustrate the transformative effect of this approach, demonstrating regulatory system strengthening, accelerated IDP implementation and maturity-level progression within a short timeframe. The CIP Network’s structured, scalable and country-driven model exemplifies how coordinated, evidence-informed and partner-aligned mechanisms can deliver sustainable improvements in regulatory performance, serving as a blueprint for global regulatory strengthening.

## Introduction

Achieving universal health coverage (UHC), as articulated in Sustainable Development Goal (SDG) 3, Target 3.8, requires equitable access to safe, effective, quality-assured and affordable essential medicines and vaccines (1). Meeting this target depends not only on expanding healthcare delivery but also on ensuring that national regulatory authorities (NRAs) have the institutional capacity to guarantee the quality, safety and efficacy of medical products. In recognition of this, the World Health Assembly (WHA) adopted Resolution 67.20 in 2014, underscoring that robust and reliable regulatory systems are foundational to resilient health systems and essential for the attainment of health-related SDGs (2).

**Figure 1:**
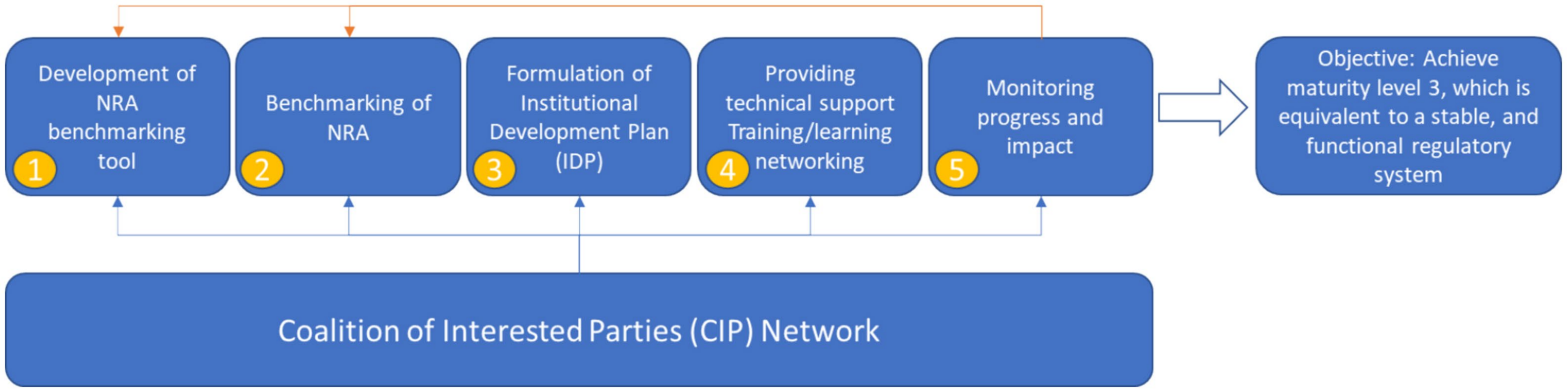
WHO five-step capacity building model (3). Sequential steps include development of the Global Benchmarking Tool (GBT), country benchmarking, formulation of institutional development plans (IDPs), provision of technical support and training, and monitoring of progress, all directed toward achieving maturity level 3 (a stable and functional regulatory system).

To address widespread capacity gaps, the World Health Organization (WHO) established the Five-Step Capacity Building Model (Figure 1), anchored by the Global Benchmarking Tool (GBT) (3). This model sets out a sequenced pathway beginning with benchmarking, followed by the development of Institutional Development Plans (IDPs), targeted technical support and training and ongoing monitoring to guide countries toward maturity level 3, a stable and functional regulatory system. Grounded in ISO 9004 quality management principles, the GBT provides a standardized methodology to assess the performance of national regulatory systems across all core functions, categorizing them from fragmented and reactive (maturity level 1) to optimized with mechanisms for continuous improvement (maturity level 4) (3, 4). Benchmarking outcomes inform the formulation of IDPs, which serve as structured roadmaps for strengthening regulatory functions through policy reform, institutional investments and technical assistance.

**Figure 2:**
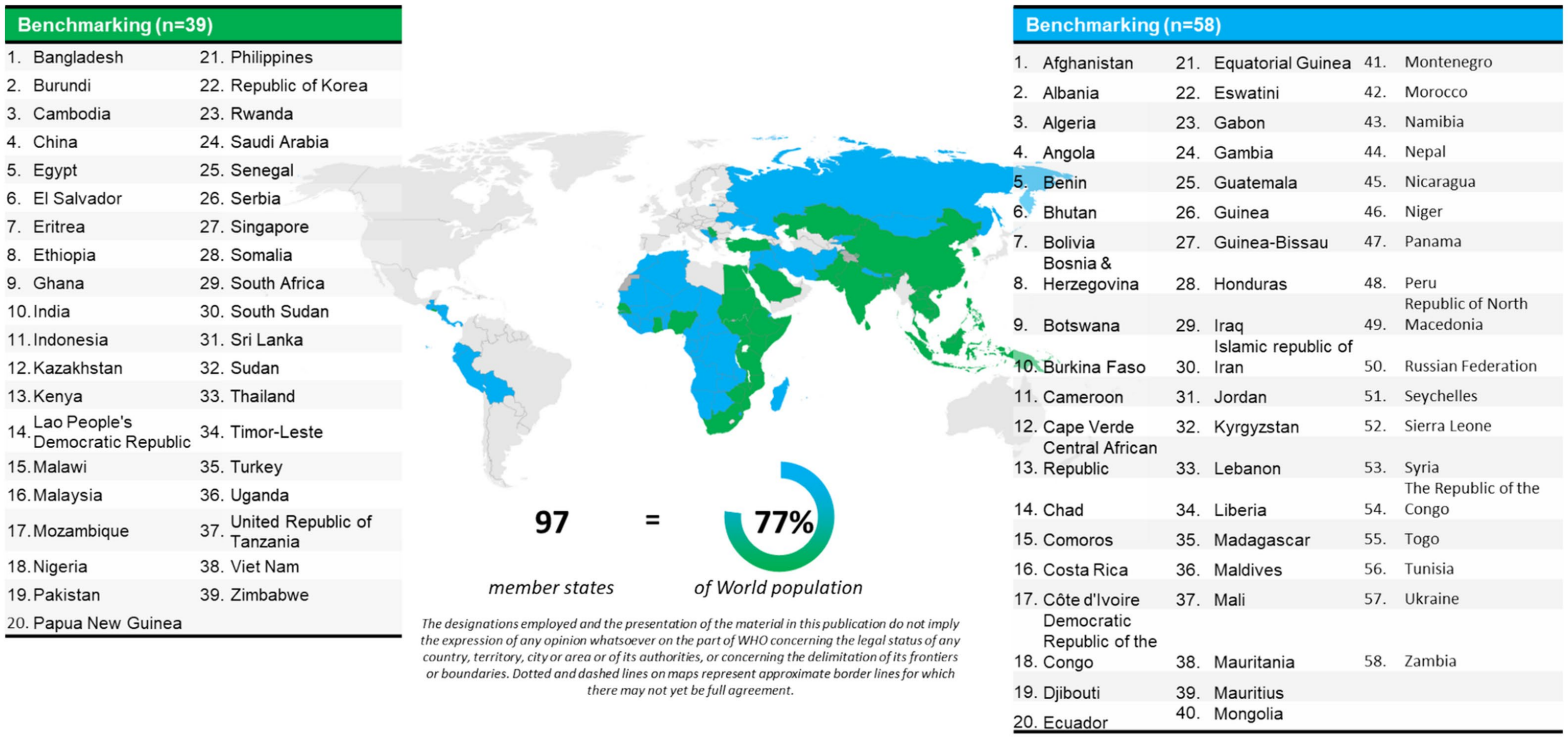
WHO member states benchmarked using the WHO global benchmarking tool (6). Map and tabulation of 97 WHO member states that have completed benchmarking exercises (formal or self-benchmarking), representing 77% of eligible countries.

**Figure 3:**
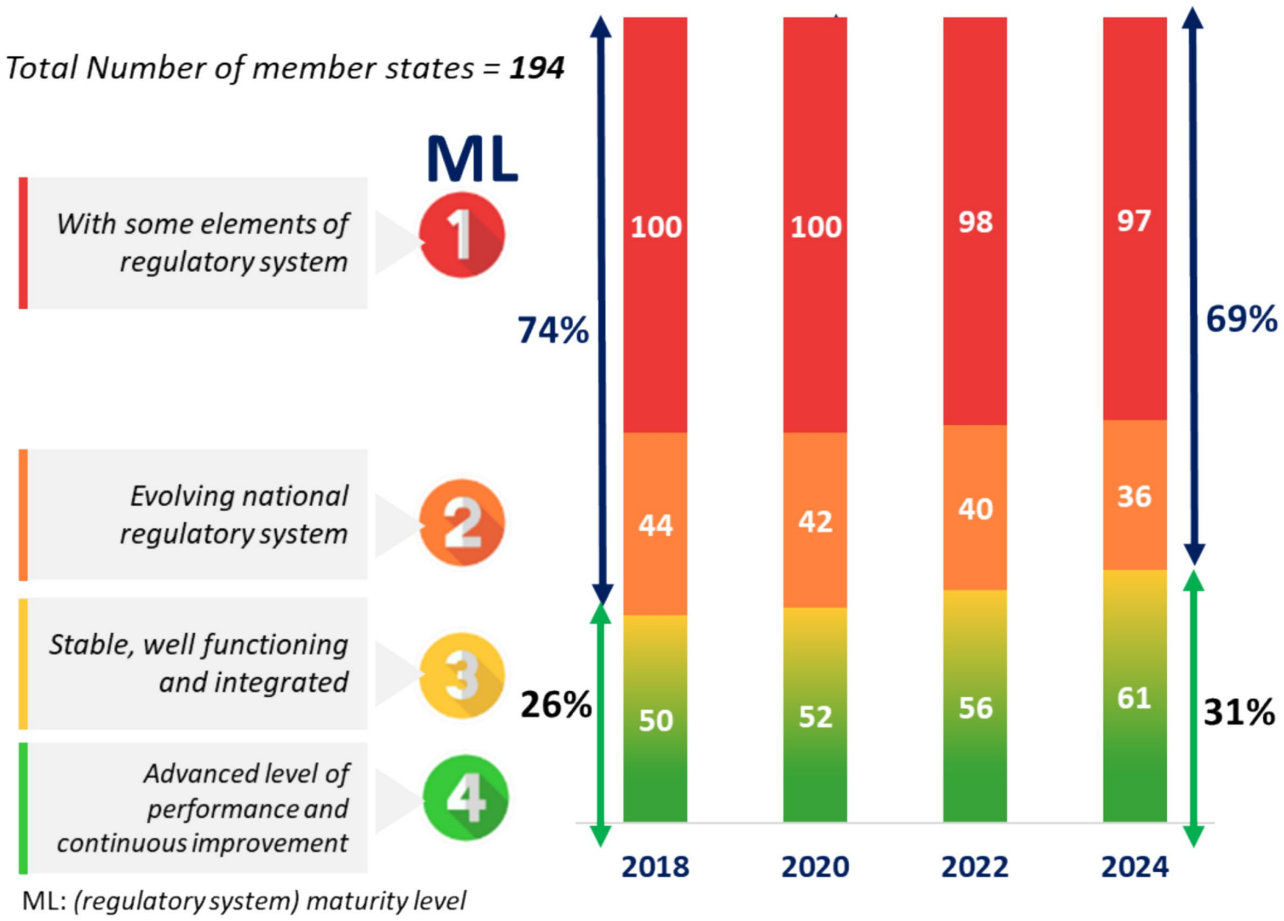
Progression of WHO member states along the regulatory maturity continuum (2018–2024) (6). Distribution of NRAs across maturity levels (ML1–ML4) showing a gradual increase in the proportion of systems at levels 3 and 4, but with the majority remaining at lower levels.

Since its introduction, the GBT has become the global reference standard for evaluating and improving regulatory systems (5). Formal benchmarking and self-assessments have been conducted in 97 Member States, representing 77% of the world population (Figure 2), highlighting both its wide application and the scale of work still required (6). Despite progress, WHO data show that nearly 70% of Member States remain at maturity levels 1 or 2, reflecting persistent structural challenges and limited capacity for sustainable reform. Between 2018 and 2024, incremental improvements were recorded, with the share of NRAs at maturity levels 3 and 4 rising from 26 to 31% (Figure 3) (6). Nonetheless, 133 Member States continue to operate at early maturity levels, emphasizing the urgency of coordinated technical and financial support. Evidence demonstrates that GBT-informed reforms can accelerate access to medicines, foster reliance among regulators and strengthen preparedness for health emergencies (7). Complementing this, the WHO-Listed Authority (WLA) framework provides a transparent mechanism for recognizing trusted NRAs, thereby facilitating reliance, improving procurement decisions, and setting performance benchmarks for others (8). Regional initiatives, such as the East African Community Medicines Regulatory Harmonization (EAC-MRH) program, illustrate how collaborative assessments can improve efficiency and consistency. However, these initiatives also highlight persistent obstacles, particularly around financing, sustainability and predictable processes, which require stronger partner coordination (9–11). Many low- and middle-income countries (LMICs), despite their commitment to reform, lack sufficient resources to fully implement improvements, underscoring the critical role of sustained external investment (12).

Strengthening regulatory systems is not simply a matter of institutional reform; it constitutes a core public health intervention directly linked to UHC and improved medicine quality outcomes (13, 14). To achieve meaningful impact, LMICs require targeted support in the form of technical expertise, policy guidance and financial resources from development partners. Such support not only helps close regulatory gaps and align systems with international standards but also facilitates local pharmaceutical production, builds public trust and advances reliance and harmonization efforts (15). In Africa, the creation of the African Medicines Agency (AMA) marks a significant policy milestone for consolidating regulatory harmonization and manufacturing ambitions, though its effectiveness will depend on sustained, coordinated backing from global and regional stakeholders (16).

Against this backdrop, the Coalition of Interested Parties (CIP) Network provides a structured platform to align and coordinate partner investments. As a central component of WHO’s Five-Step Capacity Building Model, and closely linked to the GBT, the CIP Network ensures that external contributions are transparent, targeted and responsive to country-defined priorities. By convening and coordinating diverse stakeholders under a unified governance and operating framework, the CIP Network operationalizes WHO’s normative mandate by translating the five-step model into coherent, country-led, and non-duplicative regulatory system strengthening actions (17). By translating benchmarking outcomes into coordinated action, the Network helps accelerate access to medicines, foster reliance and strengthen resilience to public health threats. Through its inclusive governance and alignment with IDPs, the CIP Network maximizes the collective impact of partner support, reinforcing both the effectiveness and long-term sustainability of WHO’s regulatory system strengthening agenda.

## Development and operationalization of the CIP Network

### Pilot implementation

Three countries, namely Bangladesh, Nepal and Rwanda, were selected by WHO to serve as pilot countries for the development and testing of the CIP Network framework. The countries were selected based on predefined criteria such as regulatory capacity, readiness to engage, active interest from multiple technical and financial partners supporting regulatory system strengthening and potential for measurable impact. The pilot methodology applied combined regulatory system assessments using the WHO GBT, in-depth stakeholder consultations and the implementation of tailored interventions, aligned with the identified priorities of each country. With thanks to the Directorate General of Drug Administration (DGDA), Bangladesh, the Department of Drug Administration (DDA), Nepal and the Rwanda Food and Drug Authority (Rwanda FDA), Rwanda, the national regulatory systems of these countries were assessed using the WHO GBT. Based on the outcomes of the assessments, the IDPs were formulated and informed the identification of the resources required for these national regulatory systems to achieve ML 3 status. The inaugural CIP Network pilot meetings were held in September 2018 (Bangladesh), January 2020 (Rwanda) and April 2020 (Nepal) in collaboration with the relevant NRAs, the WHO at the three levels (country offices, regional office and headquarters) and technical and financial partners. At the core of the discussions was the clarification of stakeholder roles and responsibilities, gaining insight into the NRAs’ priorities, the challenges faced by NRAs in implementing IDPs, and exploring mechanisms for progress monitoring, accountability and long-term sustainability.

### Lessons learned

Prior to the introduction of the GBT and the establishment of the CIP Network, regulatory strengthening efforts were often characterized by fragmented support and limited follow-through. While benchmarking and assessments were conducted, many NRAs lacked the technical, financial and human resources required to implement the resulting recommendations and IDPs. National regulatory authorities, particularly in low- and middle-income countries face significant constraints in translating assessment findings into sustained reform, due to competing routine regulatory workloads, insufficient funding and uncoordinated partner engagement (9, 12, 14). In the absence of a unified coordination mechanism, countries frequently received *ad hoc* or duplicative support, with limited alignment to national priorities or sequencing of interventions, resulting in slower and less predictable progress. A critical need had been identified for structured and well-coordinated support, as NRAs were often overwhelmed by the uncoordinated engagement of multiple technical and financial partners, frequently working in silos and without alignment to national priorities. The introduction of the GBT provided a standardized, evidence-based diagnostic framework, while the CIP Network addressed a critical gap by coordinating technical and financial partners around country-led IDPs, enabling resource mobilization, prioritization, and sustained implementation. Through the CIP Network, coordinated support efforts were initiated, aligned with the scale and complexity of partner engagement in each context. This approach responds to challenges previously identified, where regulatory strengthening efforts, despite the availability of assessment tools, were often fragmented, under-resourced and insufficiently coordinated to enable systematic implementation of benchmarking recommendations (5, 12). The collective experiences and lessons learned from the pilot implementation were instrumental in shaping the CIP Network Terms of Reference and informing the operationalization of the CIP Network. The pilot also underscored the need to move away from fragmented or wish list-driven requests for assistance, advocating instead for strategic, results-oriented investments that align with national regulatory priorities and yield measurable outcomes such as IDP implementation and maturity level progression. Similar coordination gaps have been documented in other regulatory strengthening initiatives, including continental and regional mechanisms, where the absence of structured partner alignment limited efficiency and sustainability (18). WHO played a central role as a neutral convener, providing a platform for transparent dialog and alignment of support with the country’s priority needs, based on the outcomes of WHO benchmarking activities. This neutral convening role mirrors global best practices, where WHO-anchored coordination mechanisms have been shown to enhance coherence, reduce duplication and improve the translation of assessments into action (5). Key lessons highlighted the importance of NRA leadership and ownership in the implementation of IDPs, which was essential to achieving long-term sustainability beyond the attainment of maturity level 3. It was also recognized that all support provided to countries needed to align with international norms and standards, consistent with WHO good regulatory practices and quality-management principles (3, 4). The value of targeted advocacy and sensitization had also become evident, with coordination meetings serving as an effective mechanism to foster voluntary and enthusiastic uptake of regulatory system strengthening activities among partners engaged in the pilot and to attract new potential partners. Consistent with evidence from global investment analyses, such structured coordination enables more efficient use of scarce technical and financial resources by sequencing support around nationally defined priorities rather than isolated partner mandates (12). Furthermore, the importance of providing regular updates on the implementation of committed support, evolving country needs and observed challenges and successes had been emphasized, as this contributed to the overall efficiency, transparency and impact of the CIP Network. Taken together, these features distinguish the CIP Network from other regulatory strengthening initiatives by embedding coordination directly within the WHO benchmarking and IDP cycle, thereby offering a scalable, country-owned and evidence-informed alternative to previously fragmented approaches. These combined insights laid the foundation for a structured, country-driven and partner-aligned model of support that guided the implementation of regulatory system strengthening in a more coordinated, responsive and effective manner.

### Establishment of the CIP Network

The WHO Coalition of Interested Parties (CIP) Network was formally established as a strategic initiative to enhance coordination, coherence and impact in global regulatory systems strengthening efforts. Rooted in Resolution WHA67.20 (2014), which underscored the importance of effective regulatory systems for universal health coverage and access to quality medical products, the CIP Network was launched in September 2021 following the publication of its Terms of Reference in December 2020. It was created as a voluntary WHO-led collaborative mechanism to align the growing number of technical and financial partners supporting regulatory system strengthening at country, regional and global levels. Since its establishment, the Network has operationalized key governance structures, including the Global Steering Group (GSG) in October 2022 and Regional Steering Groups (RSGs) in Africa (2022) and South-East Asia (2024). Milestones include the development of a Strategic Plan, preparation of an Operational Plan and the CIP Network Coordination Mechanism. The CIP Network has become a globally recognized platform for harmonized support guided by principles of country ownership, confidentiality, and evidence-based planning, and focused on accelerating maturity level progression and institutional development through a unified support framework.

### CIP Network terms of reference

The Terms of Reference (TOR) for the CIP Network provide a comprehensive framework for its establishment, purpose and functioning. Its core aims and objectives include optimizing the use of available resources, enhancing the consistency and sustainability of support interventions, encouraging the adoption of good regulatory practices and minimizing fragmented or duplicative efforts. The TOR are guided by key principles such as country leadership, particularly the central role of NRAs in driving the implementation of IDPs, as well as transparency, impartiality, alignment with international standards and the protection of confidential information. The governance structure includes a Global Steering Group (GSG), that sets the strategic direction and priorities of the Network, and Regional Steering Groups (RSGs), that oversee activities at the regional level; both are supported by a WHO-hosted Secretariat responsible for coordination, planning, and administrative functions. Membership is open to eligible entities including intergovernmental organizations, national authorities, non-governmental organizations, academic institutions and philanthropic foundations that meet both general and technical criteria, such as commitment to public health and demonstrated engagement in regulatory system strengthening. There are currently 33 active members, all of whom are required to sign a Confidentiality Undertaking and adhere to obligations including participation in Network meetings, ethical conduct, transparency in information sharing and alignment with WHO technical standards. CIP Network members are responsible for covering all costs and expenses relating to their participation in the Network’s governance, work and activities and the day-to-day routine operations of the Secretariat to the Network are financed by WHO. The scope of activities spans the full lifecycle of regulatory strengthening, including benchmarking using the WHO GBT, development and implementation of IDPs, coordination of technical and financial support and monitoring of progress. The TOR also mandate the use of Support Plans to structure partner engagement, define roles and responsibilities, set timelines and expected outcomes and ensure alignment with country priorities. Confidentiality and data security are safeguarded through formal consent processes and the use of a secure WHO-managed web platform, that facilitates the exchange of information and coordination among members at global, regional and country levels.

### CIP Network toolkit

The CIP Network Toolkit was developed in alignment with the requirements outlined in the CIP Network TOR, serving as the formal mechanism to operationalize collaboration between NRAs, regional regulatory networks (RRNs), WHO, CIP Network members and partners, invited by the NRA, in support of regulatory system strengthening. The toolkit comprises of four core documents. The first is the CIP Network TOR, that set out the overarching objectives, scope and governance of the CIP Network. The second component is the Support Plan, a critical tool that captures the nature and scope of collaborative activities. It maps the country’s IDP/regional workplan priorities against the technical and/or financial support committed by CIP Network members and partners invited by the NRA. It also includes clearly defined responsibilities, timelines, expected outputs and available resources, thereby serving as a key mechanism for tracking implementation and monitoring progress. The third component is the Contact List that consolidates the contact details of all key stakeholders engaged in the collaboration. This includes the head of the NRA, the NRA’s CIP Network focal point and focal points for each of the core regulatory functions. It also includes the WHO focal persons at global, regional and country levels, the focal points from each CIP Network member engaged in the country and the technical and financial partners invited by the NRA, including those who are not formal CIP Network members but are actively supporting regulatory system strengthening efforts in-country. The fourth document is the Consent Form, that formalizes the NRA’s agreement to share confidential and/or proprietary information within the CIP Network. This form is a prerequisite for enabling the secure exchange of country-specific information such as benchmarking reports, IDPs, workplans, roadmaps and other documentation necessary for coordinated support.

## Providing support through the CIP Network

Considering that the CIP Network operates as a voluntary mechanism, the Secretariat plays a facilitative role by providing visibility of country and regional support needs with all CIP Network members. This allows members to assess alignment with their own capacities and strategic priorities and to determine whether and how they can contribute to regulatory system strengthening in a given context. This approach ensures that support is both demand-driven and feasible, while promoting coordination, avoiding duplication and maximizing the impact of investments made by technical and financial partners. The process for identifying and prioritizing countries and regions for CIP Network support is non-prescriptive and is guided by a structured, three-tiered approach that emphasizes transparency, country ownership, and alignment with strategic priorities. The prioritization process is based on three key streams of information. Firstly, CIP Network members routinely provide updates on the countries or regions in which they are actively engaged, guided by their institutional mandates and priorities. This information is captured and updated at least annually in the CIP Network Support Plan and serves as the basis for mapping member activities, identifying contexts with multiple engaged stakeholders and determining opportunities for enhanced coordination. Secondly, countries undergoing benchmarking activities, using the WHO GBT, and that may benefit from targeted technical or financial support to accelerate progress toward achieving maturity level 3 or higher, are highlighted. Thirdly, NRAs or RRNs may formally request support through established WHO channels. These requests must be accompanied by written consent permitting the confidential exchange of relevant country- or region-specific information such as benchmarking reports, IDPs/regional work plans and roadmaps. In line with the recommendations of the 19th International Conference of Drug Regulatory Authorities (ICDRA), the routine sharing of this information is now encouraged as a standard prerequisite for receiving support through the CIP Network. To be considered eligible for CIP Network engagement, a country must have completed at least a self-benchmarking assessment using the WHO GBT. Additional prioritization criteria include countries earmarked for vaccine manufacturing or identified as regional technology hubs.

## CIP Network coordination mechanism

The CIP Network coordination mechanism operates across global, regional and national levels, with structured roles and processes to facilitate transparent and effective support for regulatory system strengthening. Guidance on the CIP Network coordination mechanism has been published to support further understanding and implementation of the mechanism.

### Global

The CIP Network coordination mechanism at the global level is structured around three foundational pillars: visibility, communication and accountability; each essential to advancing regulatory system strengthening through a harmonized, transparent and results-driven approach. Visibility is achieved by ensuring timely access to country and regional regulatory system strengthening priorities, that enables CIP Network members to align their technical and financial support with the most pressing needs. This pillar also ensures awareness of ongoing and planned activities across the Network, helping to identify gaps, avoid duplication and identifying synergies that leverage complementary expertise. Communication is facilitated through a suite of internal and external tools, including the secure SharePoint platform for members, the public CIP Network website, periodic circulars, coordination meetings and publications. These mechanisms ensure that all participants remain informed of progress, challenges and new opportunities, fostering a culture of collaboration and shared learning. Accountability is underpinned by the requirement for CIP Network members to provide accurate and complete information on their planned and implemented support. This information is consolidated into the annual CIP Network Support Plan, which forms the basis for tracking contributions, monitoring progress and ensuring transparency in engagements. Regular global coordination meetings are convened to review this plan and guide the collective activities of the Network. The information captured informs key performance metrics, shapes strategic decisions and strengthens the effectiveness of the Network’s coordination role at the global level. Through this integrated mechanism, the CIP Network ensures that support for NRAs and RRNs is strategically aligned, measurable and responsive to the evolving landscape of regulatory system needs.

### Regional

The CIP Network coordination mechanism at the regional level is specifically designed to support regulatory system strengthening efforts that extend beyond individual country contexts, focusing instead on the shared priorities and collaborative objectives of RRNs. The RRNs typically comprise of several NRAs that work together to enhance regulatory capacity, harmonize practices and address common regulatory challenges within a defined geographic or economic region. In this setting, the coordination process is initiated when the RRN submits a formal request for CIP Network support to WHO, along with written consent permitting the secure and confidential exchange of information relevant to the RRN’s collective activities. What distinguishes the regional approach is that the Support Plan, developed through the CIP Network Toolkit, reflects the priorities determined through consensus among participating NRAs, targeting areas where region-wide interventions or joint technical assistance are most beneficial. Rather than duplicating national efforts, the regional Support Plan serves as a strategic work plan outlining collaborative action, technical or financial inputs and expected outcomes across shared regulatory functions or initiatives. CIP Network members are invited to express interest in supporting the regional plan and RRNs may also engage non-member entities, known as invited partners, to contribute to the coordination effort. Focal points from the RRN and partner organizations are designated to oversee implementation and report on progress. The mechanism promotes collective problem-solving, efficient resource use and the scaling of good practices across borders, providing a platform for strengthened regulatory cooperation at a regional scale. The coordination process remains flexible, allowing for periodic updates to the Support Plan as priorities evolve, thereby maintaining alignment with the broader vision for regulatory convergence and institutional development within the region.

### National

At the national level, the CIP Network coordination mechanism is designed to ensure that all regulatory system strengthening support is led and owned by the NRA, in line with the guiding principles of country leadership, transparency and alignment with national priorities. Engagement begins with a formal request from the NRA, followed by a collaborative process involving CIP Network members and invited partners, formalized through the CIP Toolkit. The implementation of the collaborative process includes two distinct phases: Phase I and Phase II. In Phase I, participants are introduced to the national coordination mechanism, the CIP Toolkit and the SharePoint platform, while mapping their potential contributions against country needs. In Phase II, progress is reviewed and the NRA focal person for each regulatory function reports on the implementation status and upcoming activities. As the needs of the NRA may evolve over time, the Support Plan is treated as a living document, updated regularly in collaboration with all stakeholders. Routine monitoring and reporting are carried out by all focal points, representing the NRA, CIP Network members, invited partners and WHO at all three levels, ensuring that the implementation of the agreed activities is transparent, aligned and strategically coordinated to advance IDP implementation and strengthen national regulatory capacity.

## Impact of the CIP Network

The CIP Network has emerged as a global force multiplier for regulatory system strengthening, transforming fragmented efforts into a unified, high-impact movement. With 33 active members, including leading global donors, development partners and technical agencies, the Network has channeled collective expertise and resources toward a common goal: building stronger, more resilient regulatory systems that meet WHO standards. Between 2023 and 2025, the CIP Network mobilized more than USD 235 million in strategically targeted investments across over 50 countries and coordinated nearly 600 activities covering the full spectrum of regulatory system strengthening, including grants, training, technical assistance, study tours, workshops, twinning arrangements, human resource placements, and the development of tools, products, documents, and peer-reviewed outputs, all aimed at building sustainable regulatory capacity. This surge in coordinated support was not incidental, it is the direct outcome of a robust coordination mechanism grounded in three mutually reinforcing pillars: visibility, communication, and accountability.

By operationalizing these pillars through country-led Support Plans, a secure information-sharing platform, and regular multi-stakeholder engagements, the CIP Network mechanism has become the engine room of regulatory progress. It ensures alignment with national priorities, eliminates duplication and promotes transparency, enabling partners to identify gaps, maximize synergy and demonstrate measurable results. Importantly, CIP Network members also bring deep technical expertise across all regulatory functions and can rapidly deploy context-specific technical assistance, regardless of region or maturity level. This ensures that regulatory support is not only available but relevant, responsive and of the highest quality. The example of Rwanda exemplifies the transformative power of this model. In Rwanda, CIP Network engagement beginning in 2023 accelerated the country’s progression from WHO maturity level 1 to level 3 in just 2 years, an unprecedented leap underpinned by over 90% sub-indicator implementation and full IDP execution: demonstrating the impact of leveraging joint missions, targeted technical assistance and strategic partner alignment to achieve substantial regulatory maturity gains in record time. The CIP Network’s evolution reflects a growing recognition that collective action under a structured coordination mechanism yields exponential results. What was once scattered regulatory support has become a streamlined, data-driven and outcomes-focused movement. With over 200 activities planned in 2025 alone and more than 30 countries benefiting from technical assistance, training, grants and tool development, the CIP Network stands as a model of how high-level coordination and strategic investment can turn ambition into action and action into accelerated, measurable progress.

## Conclusion

The CIP Network has redefined how global regulatory system strengthening is conceptualized, coordinated and delivered. By shifting from fragmented, partner-led interventions to a harmonized, country-driven model, anchored in the WHO Global Benchmarking Tool, the Network has created a scalable, adaptable and results-oriented mechanism for regulatory advancement. The success seen in countries, demonstrates that when technical and financial assistance is aligned with national priorities, coordinated through structured governance and informed by validated tools, transformational change is possible, even within short timeframes. As the demand for regulatory resilience continues to grow in the face of evolving health challenges, the CIP Network offers a replicable model for sustainable impact. Its principles of visibility, communication and accountability, combined with a foundation of trust, transparency and technical rigor, position it as an indispensable mechanism for accelerating global progress toward universal access to safe, effective and quality-assured medical products.

## Data Availability

The original contributions presented in the study are included in the article/supplementary material, further inquiries can be directed to the corresponding author.

## Glossary

AMA: African Medicines Agency
CIP: Coalition of Interested Parties
DGDA: Directorate General of Drug Administration
DDA: Department of Drug Administration
EAC-MRH: East African Community Medicines Regulatory Harmonization
FDA: Food and Drug Authority
GBT: Global Benchmarking Tool
GSG: Global Steering Group
IDP: Institutional Development Plan
ICDRA: International Conference of Drug Regulatory Authorities
ISO: International Organization for Standardization
LMICs: low- and middle-income countries
ML: Maturity Level
NRA: National Regulatory Authority
RSG: Regional Steering Group
RRN: Regional Regulatory Network
RSS: Regulatory System Strengthening
SDG: Sustainable Development Goal
TOR: Terms of Reference
UHC: universal health coverage
USD: United States Dollar
WHA: World Health Assembly
WHO: World Health Organization
WLA: WHO Listed Authority
